# Molecular Targets of Fatty Acid Ethanolamides in Asthma

**DOI:** 10.3390/medicina55040087

**Published:** 2019-04-01

**Authors:** Oxana Kytikova, Tatyana Novgorodtseva, Marina Antonyuk, Yulia Denisenko, Tatyana Gvozdenko

**Affiliations:** Vladivostok Branch of Federal State Budgetary Science Institution, Far Eastern Scientific Center of Physiology and Pathology of Respiration—Institute of Medical Climatology and Rehabilitative Treatment, Russkaya st. 73g, 690105 Vladivostok, Russia; curdeal@mail.ru (T.N.); antonyukm@mail.ru (M.A.); karaman@inbox.ru (Y.D.); vfdnz@mail.ru (T.G.)

**Keywords:** fatty acid metabolism, palmitoylethanolamide, immunology, neurons, lung, inflammation, nerve growth factor

## Abstract

Asthma is a common allergic pathology of the respiratory tract that requires the study of mechanisms underlying it, due to severe forms of the disease, which are refractory to therapy. The review is devoted to the search for molecular targets of fatty acid ethanolamides in asthma, in particular palmitoylethanolamide (PEA), which has been successfully used in the treatment of chronic inflammatory and neurodegenerative diseases, in the pathogenesis of which the nervous and immune systems are involved. Recently, the potentially important role of neuro-immune interactions in the development of allergic reactions has been established. Many of the clinical symptoms accompanying allergic airway inflammation are the result of the activation of neurons in the airways, so the attention of researchers is currently focused on neuro-immune interactions, which can play an important role in asthma pathophysiology. A growing number of scientific works confirm that the key molecule in the implementation of these inter-systemic interactions is nerve growth factor (NGF). In addition to its classic role in nervous system physiology, NGF is considered as an important factor associated with the pathogenesis of allergic diseases, particularly asthma, by regulating of mast cell differentiation. In this regard, NGF can be one of the targets of PEA in asthma therapy. PEA has a biological effect on the nervous system, and affects the activation and the degranulation of mast cells.

## 1. Introduction

Asthma is a widespread chronic disease of the respiratory system, and is a significant medical and social problem throughout the world [1]. Between 1% and 18% of the population of different countries suffer from asthma, and there is a tendency to increase the number of the patients [2].

As a chronic, genetically determined, and heterogeneous inflammatory disease of the respiratory tract, asthma is characterized by reversible obstruction and bronchial hyperreactivity caused by a Th2-mediated immune response to immunological, neurogenic, physical, chemical, and environmental stimuli [3]. The majority of patients have disease symptoms that can be controlled by the combined use of inhaled corticosteroids and bronchodilators [4]. However, between 5% and 10% of patients suffer from refractory asthma [5]. Therefore, further study of the mechanisms leading to chronic inflammation and the development of bronchospasm is required to develop new and effective therapeutic approaches to the treatment of this pathology [6].

In most cases, asthma is an allergic disease, so the main mechanism of the formation of the pathological process is immune. Currently, the important role of the nervous system in the development of allergic reactions in asthma patients has been shown. Lung nociceptors are involved in the pathogenesis of airway inflammation in asthma through the bronchoconstrictor mechanism and the initiation of local neurogenic inflammation, which is the result of an imbalance of the interactions between nerve and immune cells [7]. As a result, in recent years researchers have focused on neuro-immune interactions, which can play an important role in asthma pathophysiology, due to their contribution to allergic inflammation [8]. Connections between neurons and immune cells have been found [9]. This may be explained by their proximity to the mucous membranes. The immune system activates sensory neurons through inflammatory mediators (cytokines, histamine), initiating the development of bronchospasm in asthma. Conversely, neurons interact with immune cells through neurotransmitters or neuropeptides, modulating the development of Th2-mediated immune response and the development of chronic inflammation.

The study of neuro-immune mechanisms in asthma pathophysiology will allow the finding of new therapeutic targets.

## 2. Material and Methods

Bibliographic searches were carried out in PubMed for reports of randomized controlled trials published between 2008 and 2018. In the search for publications, keyword combinations were used: “fatty acid metabolism” OR “palmitoylethanolamide” OR “immunology” OR “neurons” OR “lung” OR “inflammation” OR “nerve growth factor”.

## 3. The Role of Eosinophils and Mast Cells in the Pathophysiology of Asthma

The pathophysiology of allergic asthma is extremely multifaceted, and is characterized by chronic inflammation, which leads to a change in respiratory tract physiology manifested by the formation of hyperresponsiveness and reversible airflow obstruction [10]. Eosinophils and mast cells are cells of the immune system that are key for allergic diseases.

Eosinophils play an important role in asthma pathophysiology. These cells are the main effector cells in this disease [11]. Clinical evidence suggests that eosinophilia may be associated either with allergic forms of early asthma, which respond to inhaled corticosteroids and bronchodilators, or with severe non-allergic forms of late asthma [12].

Research data indicate the important role of mast cells in asthma pathophysiology and reveal the mechanism of their influence on pulmonary function [13]. Mast cell degranulation is accompanied by the release of allergic mediators (histamine, proteases, chemotactic factors, cytokines and arachidonic acid metabolites), which act on the vascular network, smooth muscle, connective tissue, goblet cells, and inflammatory cells of the respiratory tract, thereby causing bronchospasm [14]. In addition, mast cells are located near the main structures of the bronchial wall, such as the epithelium and smooth muscles, and their interaction is fundamental to asthma [15]. Since mast cells synthesize a wide range of pro-inflammatory mediators [16], they are involved in both the early and late stages of allergic reactions in sensitized people [17]. Constant activation of mast cells leads to chronic inflammation of the respiratory tract in asthma [18], therefore the regulation of mast cell degranulation will allow for inhibiting the release of allergic and pro-inflammatory mediators, and may be one of the important methods for the treatment of the disease.

Both mast cells and eosinophils are involved in the pathophysiological mechanisms of asthma. Despite the fact that these cells differ in a number of parameters, which determine their connection with various stages of an allergic reaction, they are always detected in chronic inflammation [19]. These cells can form an “allergic effector unit” in asthma [20]. M. Elishmereni et al. first present evidence for the relationship between mast cells and eosinophils [20]. In addition, in vitro studies conducted by the same group of authors indicate that the interaction between these cells leads to the release of pro-inflammatory mediators, which may play an important role in chronic allergic inflammation. Both activated mast cells and eosinophils can modulate each other’s functions by producing a number of mediators, such as stem cell factor, osteopontin, interleukin 5, prostaglandin D2, platelet activating factor, vascular endothelial growth factor (VEGF-A), histamine, and nerve growth factor (NGF) [21]. It can be concluded that therapeutic impact on mast cells will allow for modulating the functions of eosinophils through the interaction of specific receptors and blocking the existing interaction between mast cells and eosinophils, which plays an important role in the process of mediator release. At the same time, little is known about endogenous molecules and mechanisms modulating the functions of these cells.

## 4. The Role of Nerve Growth Factor in Asthma Pathophysiology

A number of mediators produced by mast cells and eosinophils are involved in the implementation of functions of the immune and nervous system, and also play an important role in asthma pathophysiology.

NGF is produced by both mast cells [22] and eosinophils [23]. Kim et al. have shown that NGF level correlates with the number of eosinophils, which are the main effector cells in asthma [24]. The interaction between immune cells and NGF was first found for mast cells [25]. It has been shown that NGF acts as a mast cell differentiation factor, affecting their functioning and survival by protecting against apoptosis [26]. A growing body of research on the studied problem confirms existing views that NGF is a key molecule in the interaction between the immune and nervous systems. According to the scientific literature, changes in NGF synthesis influence on both neuron physiology and immune cell activity [27]. NGF has pronounced biological action on mast and nerve cells, which is mediated by receptors expressed by these cells. Despite the fact that NGF is known primarily for its classical role in the physiology and pathology of the nervous system it level is considered as an important factor associated with the pathogenesis of allergic diseases, in particular asthma [28,29]. Using experimental animal models of asthma, Yang et al. have discovered that NGF may aggravate inflammation and airway remodeling through the Th2 immune response [30]. Besides that, under experimental conditions Qin et al. have shown the role of NGF in the Th2-mediated immune response to asthma [31]. The study of asthma pathogenesis in ovalbumin-sensitized mice provides evidence that NGF blocking decreases inflammation in the airways [32]. Shi et al. have confirmed, in an experimental asthma model, that NGF blocking inhibits airways allergic inflammation by modulating the balance of the Th1 and Th2 responses of T-cells [33]. An elevated NGF level was observed in patients with asthma after allergen bronchial provocation, and was closely related to the severity of the disease [24,34]. A high expression of NGF and tissue inhibitor of metalloproteinase-1 (TIMP-1), as well as the correlation between parameters in asthma patients, indicate a possible relationship between NGF and TIMP-1, which may play an important role in asthma pathogenesis. Renz et al. have demonstrated that NGF contributes to airway remodeling in asthma [34]. However, it is worth noting that the mechanism for the participation of NGF in the pathophysiology of chronic inflammation in asthma remains not fully understood, and is relevant for further research. It is obvious that the modulation of NGF level, which affects the activity of immune and mast cells, will allow for controlling the inter-systemic relationships in asthma. It is one of the current areas of search for approaches to the treatment of this pathology.

There is a large database of fatty acid ethanolamides, which have pronounced anti-inflammatory and analgesic activity for neuropathic pain and neurodegenerative diseases [35,36], as well as the ability to regulate the activation and degranulation of mast cells (this action is known as autacoid local injury antagonism (ALIA)) [37]. These data open prospects for the use of fatty acid ethanolamides for asthma treatment, taking into account the close relationship between the nervous and immune systems.

## 5. Palmitoylethanolamide

**Figure 1:**
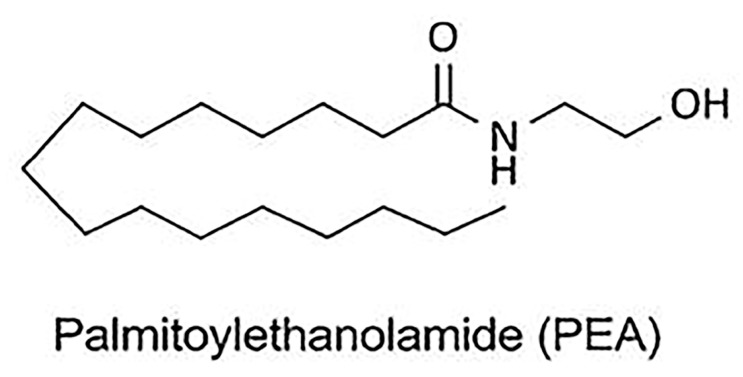
Chemical structures of palmitoylethanolamide (PEA).

Palmitoylethanolamide (N-palmitoylethanolamine, or PEA) was discovered in the late 1950s. It is an endogenous lipid mediator belonging to the class of fatty acid ethanolamides [38], acts as a signal compound, and is synthesized on demand from membrane phospholipids Figure 1. In addition, PEA is a natural lipid component found in nutritional products and food additives. It has been shown that egg yolk, peanut oil, and soy lecithin exhibit anti-allergic and anti-inflammatory properties, which are mediated by PEA fraction [39]. After studying the prophylactic and therapeutic efficacy of PEA in viral infections [40], its importance in veterinary medicine was considered (Redonyl) and then it was described as a nutritional supplement for the treatment of chronic pain and chronic inflammation (Normast, Pelvilen, Peapure, Epitech Srl, and Recoclix) [41]. At present, there is overwhelming evidence that PEA metabolism is disturbed in inflammatory, neurodegenerative diseases and pain syndromes [36]. Recent data obtained by Portavella et al. confirm that PEA has a neuroprotective effect [42]. It allows considering PEA as an anti-inflammatory, neuroprotective and analgesic mediator, and opens new prospects for its use [43]. Although the biological effects of PEA are well documented, the molecular mechanisms and targets for its action remain a subject for discussion. The most studied organ-targets are the intestine [44], the bladder [45], the skin [46], kidneys [47], and the central nervous system [48]. At the same time, the possible effect of PEA on the respiratory system has been studied in singular works [49].

## 6. Molecular Targets and the Mechanisms of Action of Palmitoylethanolamide

**Figure 2:**
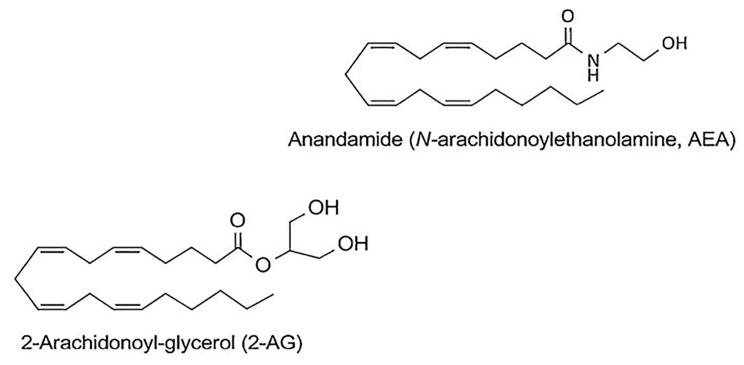
Chemical structures of N-arachidonoylethanolamine or anandamide (AEA), and 2-arachidonoylethanolamine (2-AG).

**Figure 3:**
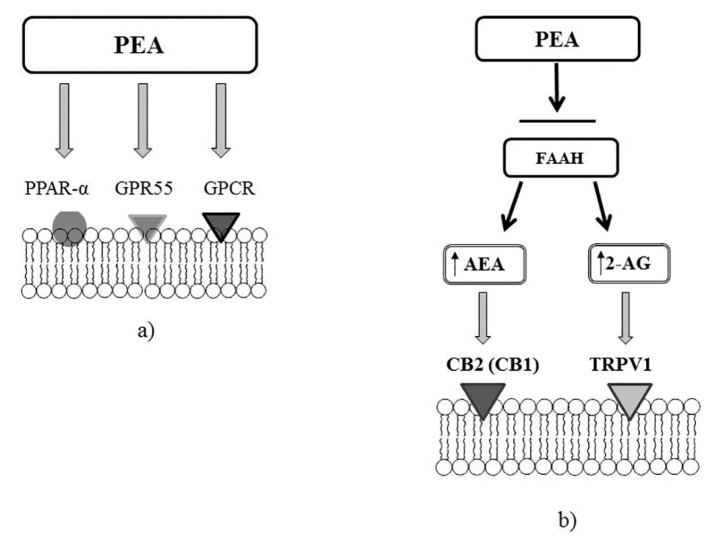
Molecular targets of PEA. (**a**) PEA can directly activate G protein-coupled receptor α (PPARα) or a cannabinoid receptor G protein-coupled receptor (GPR55). (**b**) PEA through the inhibition of the expression of fatty acid amide hydrolase (FAAH), may increase the endogenous levels of AEA and 2-AG, which directly activate cannabinoid receptor 2 (CB2) (or cannabinoid receptor 1, CB1) and transient receptor potential vanilloid receptor 1 (TRPV1) channels.

PEA is a structural analogue of anandamide (AEA), and has similarity to the ligands of N-acylethanolamine (NAE) of cannabinoid receptors (Figure 2). The direct or indirect (through action on endogenous ligands or through receptor expression) molecular targets of PEA are G protein-coupled receptors (GPCR), as well as cannabinoid receptors 1 (CB1) and 2 (CB2) belonging to their family; a new cannabinoid receptor orphan G protein-coupled receptor (GPR 55); transient receptor potential (TRP) ion channels, in particular TRP vanilloid receptor 1 (TRPV1); nuclear peroxisome proliferator-activated receptors (PPARα/γ/δ); and ATP-sensitive potassium channels (K_ATP_ channel) [50] Figure 3.

PEA has low affinity for CB1 and CB2 receptors; as a result, this lipid mediator realizes its biological effect through the endocannabinoid 2-arachidonoylglycerol (2-AG), which is the endogenous ligand of CB2 receptors [36] (see Figure 2). PEA acts as an endogenous CB2 cannabinoid receptor agonist under in vitro conditions. The analgesic effect of PEA is inhibited by CB2 antagonist -SR144528 under in vivo conditions [51]. It has been shown that PEA increases 2-AG level two times [46], under both in vitro and in vivo conditions [52]. There are findings that indicate that 2-AG is an agonist for TRPV1 channel [36]. TRPV1 desensitization is the basis of the analgesic and anti-inflammatory effects of TRPV1 agonists, which currently are used to treat neuropathic pain [53]. In addition, there are indications that PEA can significantly enhance TRPV1 activation under exposure to 2-AG in vitro [52]. These results have allowed the uncovering of another mechanism of action for this lipid mediator, opening new prospects for the realization of powerful, TRPV1-mediated anesthetic and anti-inflammatory action. Thus, the sensitivity of nociceptors, the number of which significantly increases in the lungs of asthma patients, is associated with the activity of sensory neural TRP ion channels.

NGF is a stimulus for the development of a prolonged pain syndrome. It is known that its level rises in chronic pain conditions. Recent studies have shown that NGF can modulate TRPV1 activity in rats [54]. Eskander et al., have established that NGF causes a stable, nociceptive state mediated by TRPV1 activity and oxidative mechanisms [55]. Consequently, PEA can have an analgesic effect by influencing TRPV1 activity and the NGF level, which mediate the development of a prolonged pain syndrome. 

The multifaceted biological effects of PEA are also mediated by its interaction with receptors GPR55 [56], GPR119 [57], and PPARα [51]. 

There are three isoforms of PPAR: PPARα (NR1C1), PPARβ/δ (NR1C2), and PPARγ (NR1C3). Peroxisome proliferator-activated receptors can be activated by different types of fatty acids (FA) that are part of the PAE molecule. Linolenic acid (C18:2 *n*-6; LA) and alpha-linolenic acid (C18:3 *n*-3, ALA), which are long-chain polyunsaturated FAs (LCPUFAs) and play a key role in bronchopulmonary diseases, have the highest affinity for PPAR.

PPARα is expressed in tissues with a high FA oxidation and is involved in cell proliferation, apoptosis, and differentiation, as well as the normalization of the balance between lipid peroxidation and antioxidant protection by increasing the activity of antioxidant enzymes. PPARα activation by LCPUFA (mainly C18:3 *n*-3, or ALA) appears to play a key role in the prevention and treatment of asthma. The action of PEA via PPARα leads to the activation of the TRPV1 channel [58]. Thus, the activation of the PPARα receptor also mediates the anti-inflammatory and analgesic effects of PEA [47]. In addition, the activation of two other isoforms of the PPAR receptor family, including PPARβ/δ and PPARγ, which initiates peroxisome proliferation [59], is not excluded. PPARα impacts fatty acid metabolism, and its activation reduces lipid levels, whereas PPARγ is involved in the regulation of adipogenesis, energy balance, and lipid biosynthesis. PPARβ and PPARδ are involved in the oxidation of fatty acids and the regulation of glucose and cholesterol levels in the blood.

As noted above, NGF level mediated by TRPV1 activity increases in chronic pain conditions [55]. In turn, PEA action is mediated through 2-AG, which acts as an agonist of the TRPV1 channel [36]. These facts indicate the possibility of the realization of analgesic and anti-inflammatory effects of PEA-mediated TRPV1.

The analgesic effect of PEA has been discussed by Hesselink [60].

## 7. Palmitoylethanolamide and Asthma

NGF is considered in close connection with asthma pathogenesis [29]. At the same time, there is evidence of the simultaneous increase in the number of mast cells and the NGF level in chronic inflammation [61]. These indicators also play an important role in asthma pathophysiology [13,14]. Taking into account the targets for biological action of PEA, we can assume that PEA can contribute to the regulation of the NGF level, which is elevated in chronic bronchopulmonary diseases. In addition, a decrease in the activity of mast cells by regulating the NGF level will affect the interaction between mast cells and eosinophils, which results in the release of soluble mediators in chronic allergic inflammation [20].

The existing view of the potentially significant role of PEA in the airways is confirmed by the high expression of this lipid mediator in the bronchi (6 nmol/g), which is about ten times higher than its level in the brain of mouse (100–550 pmol/g) [47]. Besides that, all molecular targets of PEA (PPARα, GRP55, TRPV1, CB1, and CB2) are localized in the bronchi of mice, which suggests an extremely important role of PEA in the physiology and the pathology of the lungs [36]. Zhou et al. have detected a change in CB2 and GPR55 levels in sensitized mice [62]. Perhaps the activation of these receptors is associated with increased mast cell infiltration. The research of the Nobel Laureate R. Levi-Montalcini have demonstrated the importance of inactivating the inflammatory processes through the influence of PEA on mast cells [63]. According to Calignano et al., PEA reduces the migration and the degranulation of mast cells [64]. Current scientific literature suggests that the endogenous lipid mediator PEA, which is chemically bound to the endogenous cannabinoid anandamide and acts as the local inhibitor of mast cell inflammation [65]. Facci et al. have shown that PEA suppresses cell degranulation mediated by the CB2 cannabinoid receptor [66]. Therefore, CB2 receptors may be involved in the mechanism of the action of PEA on mast cells. The importance of GPRR55 for the most of the inhibitory effects of PEA on activated mast cells has been shown [67]. Additionally, Rinne et al., have revealed that PEA increases the expression of proto-oncogene tyrosine protein kinase (Mer tyrosine kinase (Mer)) by activating GPR55, and therefore affects the function of macrophages [68]. The possible contribution of PEA to allergic sensitization has been investigated in the study of Roviezzo et al. [69]. This research group was the first to describe a decrease in bronchial level of PEA in mice with allergic reactions (after the sensitization of allergens). A reduction in the regulation of PEA expression is specific for the bronchi, since no differences in plasma level of PEA between control and sensitized mice were detected. A decrease in the PEA level in sensitized mice is a loss of the endogenous anti-inflammatory mechanism, in light of the known PEA-mediated mechanism (ALIA). This hypothesis is supported by other studies demonstrating the therapeutic effect of some anti-inflammatory drugs associated with the restoration of the endogenous level of PEA [70]. In particular, Di Paola et al. has found that the use of ultra-micronized palmitoylethanolamide (PEA-um) reduces the severity of inflammation in the lungs in a mouse model of idiopathic pulmonary fibrosis [49].

Thus, based on the relationship between the nervous and immune systems, and existing data on the mechanisms of the biological action of PEA and its role in asthma, we can talk about the prospects for the use of fatty acid ethanolamides, in particular PEA, in asthma therapy.

## 8. Conclusions

Allergic airway inflammation is associated with complex neuro-immune interactions that can play a crucial role in chronic inflammation and asthma progression. Although immune-mediated therapy for asthma has shown some success, patients with severe forms of the disease are often resistant to this treatment. Thus, the nervous system and neuro-immune interrelations may become a new and promising therapeutic target in asthma, in particular its allergic form.

A key molecule mediating neuro-immune interactions is NGF. In addition to its classic role in the pathophysiology of the nervous system, NGF is considered to be an important factor associated with the pathogenesis of allergic diseases, particularly asthma, due to its influence on the differentiation of mast cells. In this regard, NGF can be one of the targets for the action of PEA, which has a proven biological effect on the nervous system, as well as on the activation and the degranulation of mast cells. These data open prospects for the use of fatty acid ethanolamides in asthma treatment, taking into account the close relationship between the nervous and immune systems.
